# Development and validation of a spectrophotometric method for the quantification of total bufadienolides in samples of toad glandular secretions

**DOI:** 10.1590/1678-9199-JVATITD-2024-0064

**Published:** 2025-05-16

**Authors:** Elcio Daniel Sousa Barros, Evaldo dos Santos Monção, Mariana Helena Chaves, Cícero Alves Lopes, Gerardo Magela Vieira

**Affiliations:** 1 Laboratory of Natural Products, Department of Chemistry, Federal University of Piauí (UFPI), Teresina, PI, Brazil.; 2 Department of Teaching, Research and Extension, Federal Institute of Maranhão (IFMA), Coelho Neto, MA, Brazil.; 3 Bioanalytics Study Group, Department of Chemistry, Federal University of Piauí (UFPI), Teresina, PI, Brazil.

**Keywords:** Glandular secretion, Bufadienolides, *Rhinella* genus, UV-Vis method, Quantification

## Abstract

**Background:**

Bufadienolides are the main secondary metabolites found in the paratoid gland secretions (PGS) of toads of the Bufonidae family. These compounds are considered the main bioactive components of PGS. The aim of this study was to develop and validate the first method for the quantification of total bufadienolides (free and esterified) in samples of paratoid secretions from toads, using the UV-Vis absorption spectrophotometry technique.

**Methods:**

The proposed method was based on the bathochromic shift induced by the reaction of the α-pyrone group of bufadienolides (296 nm) with a 5% (w:v) aqueous solution of sodium hydroxide and detection at 356 nm, after 60 min (time defined based on the evaluation of kinetic assays).

**Results:**

The proposed method showed wide linearity (r = 0.9999), low LOD (1.3 × 10^-4^ µg/mL) and LOQ (3.9 × 10^-4^ µg/mL), recovery (84%-99%), repeatability (%RSD ≤ 5), reproducibility and robustness (p > 0.05). The total bufadienolide content in PGS extracts from 12 samples of *R. diptycha* ranged from 478 to 801 mg of EqMB/g of extract, while the *R. granulosa* sample presented 661 mg of EqMB/g of extract.

**Conclusion:**

The new developed method is innovative, simple, fast, accurate, robust, low cost, and can contribute to future research focused on the quantification of total bufadienolides in samples of toad glandular secretions. In addition to serving as a strategic tool in the selection of work matrices, optimizing time, and minimizing costs.

## Background

Toads are amphibians belonging to the order Anura, with a wide global distribution occurring most frequently in regions with humid and tropical climates [1, 2]. These animals are considered the most diversified anurans globally, exhibiting a high degree of morphological and behavioral variety. For this reason, they are considered potential bio-indicators of environmental quality and exhibit dry skin with a rough appearance and short limbs lacking interdigital membranes [3]. They are essentially terrestrial individuals with nocturnal habits and are physically larger when compared to other anurans (frogs and tree frogs) [4,5].

In the dorsolateral region of the head, these animals present a pair of paratoid glands that store highly toxic secretion with varied chemical composition. These secretions serve the purpose of defense against infections, microorganisms, and predators [6, 7]. Biochemical and pharmacological assays revealed that although the paratoid secretions of different toad species exhibit similar chemical profiles, it is possible to observe some specificities that are directly associated with phylogenetic differences, genus, age, diet, defense strategy, seasonal variations, and the diversity of habitats in which the animals live [8, 9]. The paratoid gland secretions (PGS) produced by toads are rich in biologically active components and exhibit a cardiotonic effect, primarily composed of alkaloids, arginine diacids, bufadienolides, and bufotoxins [10, 11].

**Figure 1.**
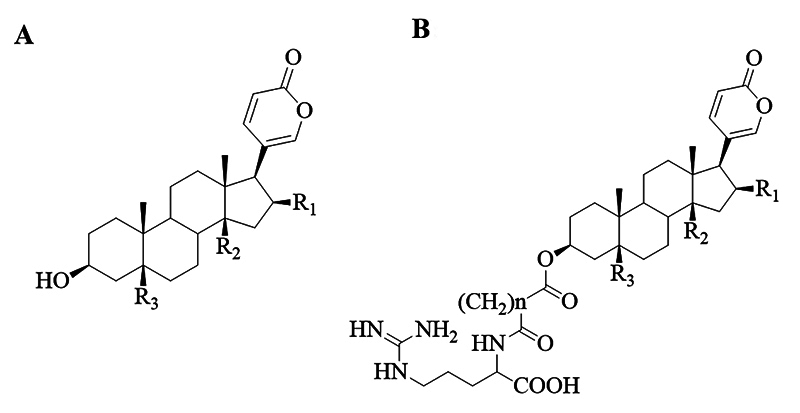
Basic structure of bufadienolides **(A)** in the free form (bufogenin) and **(B)** in the esterified form (bufotoxin).

Bufadienolides are polyhydroxylated compounds with 24 carbons, characterized by featuring the α-pyrone group attached at the C-17 position of the cyclopentanoperhydrophenanthrene system of the steroid nucleus. These metabolites have cholesterol as a biosynthetic precursor and are found in animals, primarily in amphibians, and some plant families [12, 13]. In anurans, they may appear in free form (bufogenins) or as esters (bufotoxins) when conjugated at position C−3 with carboxylic diacids linked to an amino acid, usually arginine derivatives [14] (Figure 1). The main substituents groups (R) in bufadienolides are hydroxyl, ester, epoxide, ketone, among others. Studies indicate that the presence of these substituents can either increase or decrease the biological potential of bufadienolides, but they do not alter wavelength of the chromophore (α-pyrone ring) these metabolites [15].

The biological action of bufadienolides is primarily targeted at the heart and involves the inhibition of the Na^+^/K^+^-ATPase enzyme, resulting in a positive chronotropic and inotropic effect, wherein high doses can lead to cardiac arrests [16]. In addition to their cardiac effects, they function as local anesthetics and exhibit various other biological activities, such as antiviral, cytotoxic, antibacterial, antiparasitic, insecticidal, antiangiogenic, hypertensive, and immunosuppressive effects [17-22]. These metabolites are regarded as the primary bioactive compounds found in the paratoid secretion of toads belonging to the Bufonidae family [23].

The quantification of bufadienolides in glandular secretions extracts from toads is primarily performed by HPLC-UV/DAD. However, existing chromatographic methods can’t determine the concentration of total bufadienolides in the samples, limiting themselves to the specific quantification of metabolites [24]. Thus, the present study aimed to develop and validate the first method for the quantification of total bufadienolides (free and esterified) in samples of paratoid secretions from toads, using the UV-Vis absorption spectrophotometry technique.

## Methods

### General experimental procedures

The UV-Vis spectra were acquired using a Thermo Scientific Genesys 10S spectrophotometer, with optical path length of 1 cm, scan speed of up to 3600 nm/min, and data resolution of 1 nm. The purification of the standard by semi-preparative high-performance liquid chromatography, reverse-phase, was carried using a Shimadzu^®^ prominence system chromatograph equipped with binary pump system LC-6AD, manual injector, UV detector SPD-20A, automatic collector, and Phenomenex Luna C18 column (250 × 10 mm, 10 µm).

The purity of the obtained standard was assessed using a Shimadzu chromatograph equipped with DGU-20A degassing unit, LC-20AT pump system, SIL-20AHT automatic injector, CTO-20A column oven, SPD-M20A diode array detector, CBM-20A communication module, and Phenomenex Luna C18 column (250 × 4.6 mm, particle size 5 μm). LC-MS analyses were performed using high-performance liquid chromatography (HPLC − Shimadzu LC-6AD) coupled to a mass spectrometer (micrOTOF QII, Bruker Daltonics) with electrospray ionization (ESI) coupled to a high-resolution quadrupole. Chromatographic analysis was performed on a Kinetex® XB-C18 column (100 × 2.1 mm, particle size 2.6 µm, Phenomenex).

In the high-performance chromatographic analyses, HPLC grade solvents from J. T. Baker and ultrapure water (≥ 18 MΩ·cm) obtained from Milli-Q Plus system and Master All ultrapurification system from Gehaka were used. The extracts were prepared using analytical grade solvents from Synth, with purity of 99.5%; Cristófoli ultrasonic bath with capacity of 2 L and ultrasonic frequency of 42 kHz; and a Heidolph Laborota 4000 rotary evaporator operating at 60 rpm and 40 °C, equipped with vacuum pump with oil compressor from Prismatec, model 131, type 2 VC, and 1/4 HP single-phase motor of 60 Hz.

### Collection of paratoid secretions

The toads of the species *Rhinella diptycha* and *Rhinella granulosa* were identified by biologists from the Federal University of Piauí, Picos campus (Brazil), under the supervision of professor and herpetologist Dr. Mariluce Gonçalves Fonseca (IBAMA/SISBIO n^o^. 22508-2). The biological secretion of interest (PGS) was collected in the toad’s natural habitat by manually compressing of the paratoid glands of the animals (average of 10 animals per collection). After extraction of paratoid secretions, the animals were safely returned to their natural habitat without any injuries or harm.

The paratoid secretions of R. diptycha toads were collected, considering gender (male and female), in the cities of Picos (7°04'48"S 41°26'10"W − southern region of the state of Piauí, Brazil), Teresina (5°02'53"S 42°47'02"W − central region of the state of Piauí) and Parnaíba (2°50'35”S 41°45'38”W − northern region of the state of Piauí), during the months of February (rainy season) and November (dry season) of 2021, resulting in 12 samples of the species.

In contrast, due to difficulties in locating individuals, the small number of captured specimens, the discreet size of the parotoid glands, and consequently, the challenges in extracting secretions, as well as the limited amount of glandular material obtained from *R. granulosa*, the paratoid secretions of this species were collected without distinguishing gender (male or female), only in the city of Picos and exclusively in February (rainy season) of 2022.

Regarding the captured specimens, the females exhibited a black-colored dorsum with light spots (brown or yellow), a slightly larger size compared to males (which had a yellowish coloration without a dark dorsum), and the absence of a nuptial pad and vocal sac (organs exclusive to male toads). At the end of collections, a total of 13 samples of PGS were obtained.

The collection of PGS was conducted after obtaining the scientific research registration (SISGEN n^o^. AE58A09), acquiring the permanent license for the collection of zoological material (IBAMA/SISBIO n^o^. 55970-1), and receiving approval from the Ethics Committee for Animal Use of the Federal University of Piauí (CEUA/UFPI n^o^. 52107-2). Voucher specimens (*Rhinella diptycha* - CHCJ#0669 and *Rhinella granulosa* - CHCJ#007) were deposited in the Herpetology Scientific Collection Jorge Jim (CHCJ) at the Federal University of Piauí, Picos campus.

### Preparation of PGS extracts

During the collection, the paratoid secretion of each animal was placed in disposable plastic containers and subsequently stored in a desiccator with silica for 72 h at room temperature under vacuum. After this period, the dried PGS was transferred to glass containers and stored in the freezer at 4 °C. The extracts were prepared by adding 50 mL of methanol to 1 g of powdered PGS. The mixture was sonicated in an ultrasonic bath for 15 min (four cycles), followed by simple filtration. After rotary evaporation of the excess methanol, a total of 13 PGS extracts were obtained: twelve from *R. diptycha* (E01-E12) and one from *R. granulosa* (E13).

The identification of the extracts was carried out based on the species, gender, seasonal period, and the city of paratoid secretion collection. The PGS from *R. granulosa* was collected without gender distinction. Thus, the extracts were designated as follows: E01 - Diptycha Female Rainy Parnaíba; E02 - Diptycha Male Rainy Parnaíba; E03 - Diptycha Female Dry Parnaíba; E04 - Diptycha Male Dry Parnaíba; E05 - Diptycha Female Rainy Teresina; E06 - Diptycha Male Rainy Teresina; E07 - Diptycha Female Dry Teresina; E08 - Diptycha Male Dry Teresina; E09 - Diptycha Female Rainy Picos; E10 - Diptycha Male Rainy Picos; E11 - Diptycha Female Dry Picos; E12 - Diptycha Male Dry Picos; and E13 - Granulosa Rainy Picos.

### Purification of the marinobufagin standard

The bufadienolide marinobufagin was used as a standard substance in the experimental assays. The compound was isolated from the ethyl acetate extract of the paratoid secretions of *R. diptycha* toads (EARD) after chromatographic fractionation on a silica gel column, using as mobile phase, the mixture of chloroform and methanol solvents, in increasing order of polarity, followed by semi-preparative high-performance liquid chromatography. The EARD extract was prepared by adding 150 mL of ethyl acetate (AcOEt) to 20 g of powdered paratoid secretion sample from *R. diptycha*. The mixture underwent sonication in an ultrasonic bath for 30 minutes, followed by simple filtration, for four cycles. After evaporating the excess solvent using a rotary evaporator under reduced pressure at 40 ºC, the extract was obtained (460 mg). The purified marinobufagin was analyzed by thin layer chromatography (TLC), HPLC-DAD and HPLC-HRMS, being identified by comparison with data reported in the literature, considering the *m/z* of the protonated molecule ion (HRMS) and the calculated relative error [25-27]. Chromatographic information regarding the isolated marinobufagin is presented in Additional files (Additional files 1-5).

The high-performance semi-preparative liquid chromatography separation was carried out using a mobile phase consisting of H2O (solvent A) and MeCN (isocratic system − 40% of solvent B), with flow rate of 4 mL/min, injection volume of 300 µL, and detection at λ = 296 nm. The analysis conditions for verification of the purity of the isolated marinobufagin, in HPLC-DAD, included an exploratory gradient with mobile phase composed of ultrapure water (solvent A) and acetonitrile (solvent B) in the following proportions: 5% to 100% of solvent B over a time of 0.1 to 35 min. The injection volume was 10 μL, with flow rate of 1.0 mL/min and monitoring at 296 nm.

LC-MS analysis was conducted in the following conditions: mobile phase composed of water + 0.1% formic acid (A) and acetonitrile + 0.1% formic acid (B), flow rate of 1 mL/min, injection volume of 10 µL, and the oven temperature fixed at 30 ºC. A linear elution gradient from 10% to 100% mobile phase B was used over 15 minutes, followed by an isocratic gradient for 3 minutes. Mass spectrometry data were recorded in positive mode, within the range of *m/z 150−1200. Parameters used in the ionization source were capillary voltage of 3500 V, drying gas flow of 9* L/min, drying gas temperature of 250 °C and nebulizer gas pressure of 4.5 Bar. Nitrogen was used as nebulizing, drying, and collision gas.

The chromatographic plate (CCD) of the marinobufagin, eluted in a solvent system consisting of chloroform/methanol (95:5) and revealed on a heating plate (≈ 90 ºC) after spraying with a solution of p-anisaldehyde-sulfuric acid, displayed a single brown spot, indicating the isolated nature of the compound (Additional file 1). Additionally, the HPLC-DAD chromatogram of the investigated marinobufagin showed a single peak (tR = 12.88 min) at a wavelength set to 296 nm (the maximum absorption of bufadienolides) and exhibited chromatographic purity of 95% (Additional files 2 and 3).

### Development of the UV-Vis spectrophotometric method

The proposed method for the quantification of total bufadienolides in samples of toad glandular secretions, using the UV-Vis absorption spectrophotometry technique, was based on the bathochromic shift induced by the reaction of the α-pyrone group of bufadienolides (296 nm), present in the methanolic extracts of the paratoid secretion samples collected, with a 5% (w:v) sodium hydroxide solution, and detection at 356 nm after 60 min (defined through assessment of kinetic assay). The spectrophotometric analysis was carried out in triplicate using 100 µL of PGS extract solution from toad (25 µg/mL), 100 µL of sodium hydroxide 5% solution, and 1800 µL of methanol in quartz cuvettes.

### Construction of the analytical curve for total bufadienolides quantification

Initially, an aqueous solution of 5% sodium hydroxide (w:v) and three stock solutions of the marinobufagin (MB) standard in methanol (100 µg/mL) were prepared. In Falcon tubes, eight working solutions of the MB standard were prepared (2.5, 5.0, 7.5, 10.0, 12.5, 15.0, 17.5, and 20.0 µg/mL) by mixing 100 μL of 5% aqueous sodium hydroxide solution with increasing volumes of the stock standard solution, adjusting the final mixture volume (2000 μL) with methanol. After preparation, the working solutions were subjected to mechanical agitation in a Kasvi basic vortex mixer for 3 minutes at room temperature, followed by resting for an additional 57 minutes. The absorbance values of the mixtures were recorded in triplicate after 60 minutes of reaction at a wavelength of 356 nm. The analytical curve for quantification of total bufadienolides was constructed by correlating the obtained absorbance values with the concentrations of the marinobufagin standard working solutions.

### Validation of the developed analytical method

The validation of the proposed method was carried out following the recommendations of the International Conference on Harmonization (ICH), based on the assessment of the following performance parameters: selectivity, linearity, limits of detection (LOD) and quantification (LOQ), precision, accuracy, and robustness [28]. The selectivity of the method was assessed by comparing the UV-Vis spectra of the investigated extracts before and after reacting with the 5% sodium hydroxide solution. The linearity was determined by the correlation coefficient (r) of the analytical curve constructed from the values obtained from the mean absorbances recorded after the reaction of the reference standard marinobufagin, at eight different concentrations (2.5-20.0 µg/mL), with the 5% aqueous NaOH solution. The minimum amount of the analyte that can be detected by the method Limit of Detection (LOD) and the minimum amount of the analyte that can be quantified Limit of Quantification (LOQ) defined, based on the values of the analytical curve slope (S) and standard deviation of the intercept (s), were calculated according to Equations 1 and 2.

$$LOD=3.3\;\times \;\frac{s}{S}$$ (Equation 1)

$$LOQ=10\;\times \;\frac{s}{S}$$ (Equation 2)

The precision of the method was verified through repeatability (intraday precision) and reproducibility (interday precision) of the spectrophotometric analyses at the same concentration (25 µg/mL). Intraday precision was analyzed by performing eight analyses on the same day, while interday precision was evaluated by conducting sixteen analyses in two consecutive days (eight readings each). After the analyses and determination of the total bufadienolides contents in the samples, the relative standard deviations (%RSD) for the obtained datasets were calculated to assess the precision (intraday and interday) of the method. The accuracy of the method was evaluated using the standard recovery assay. Initially, three distinct solutions of the marinobufagin standard were prepared (50 - 100 - 200 µg/mL). Then, the spiked extract solutions (standard addition) were prepared from a mixture of 100 µL of the extract solution, 100 µL of the standard solution, 1700 µL of methanol, and 100 µL of the 5% sodium hydroxide solution. The reagent mixture was subjected to mechanical agitation (3 min) and, after 60 min, was analyzed using a spectrophotometer (356 nm). After spectrophotometric analysis and measuring of the absorbances, the mean concentration of the standard calculated in each extract solution (spiked and non-spiked) was determined using the analytical curve. All analyses were performed in triplicate, and the percentage of standard recovery was calculated using Equation 3.

$$\%\;Recovery=\left(\frac{CD-CB\;}{CT}\right)\;x\;100$$ (Equation 3)

where: CD is the concentration of the standard calculated in the doped sample (extract + standard); CB is the concentration of the standard calculated in the undoped sample (extract only) - blank of the analysis; and CT is the theoretical concentration of the added standard in the extract solution. The robustness of the method was assessed by comparing the means obtained for the concentration of total bufadienolides in the samples defined from spectrophotometric analyses performed on extract solutions with the proposed conditions and extract solutions with alteration to one of the parameters defined by the developed method (t-test). The modified parameters were: reaction and agitation times of the reagent mixture, base volume, base concentration, and substitution of the base used (Table 1).

**Table 1.** Experimental parameters analyzed in the evaluation of the robustness of the proposed method.

Parameter	Proposed condition	Variation	Test
Base volume	100 µL	125 µL	T1
Base concentration	5%	6%	T2
Used base	NaOH	KOH	T3
Reaction time	60 min	65 min	T4
Agitation time	3 min	1 min	T5

### Determination of the total bufadienolides content in PGS extracts

The total bufadienolides content in the investigated extracts was determined after reacting the respective sample solutions (25 µg/mL) with a 5% sodium hydroxide solution, following the conditions outlined in the proposed method. Based on the recorded absorbances and the constructed analytical curve, it was possible to determine the total bufadienolides content in the extracts, with the concentration value expressed in milligrams of marinobufagin (MB) equivalents per gram of extract (mg of EqMB/g of extract) ± standard deviation.

## Results

### Validation of the developed UV-Vis spectrophotometric method

**Figure 2.**
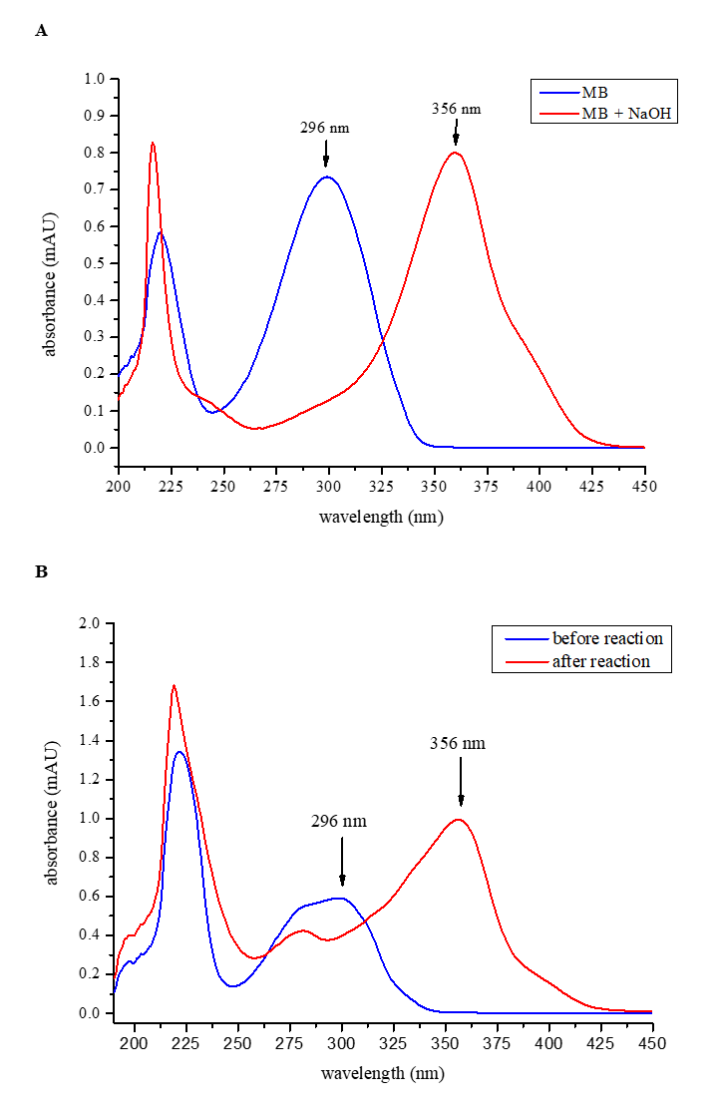
UV-Vis spectrum showing the bathochromic shift induced after reaction of the 5% NaOH solution in excess with **(A)** marinobufagin standard (MB) and **(B)** extract solution of the investigated parotoid gland secretions.

**Figure 3.**
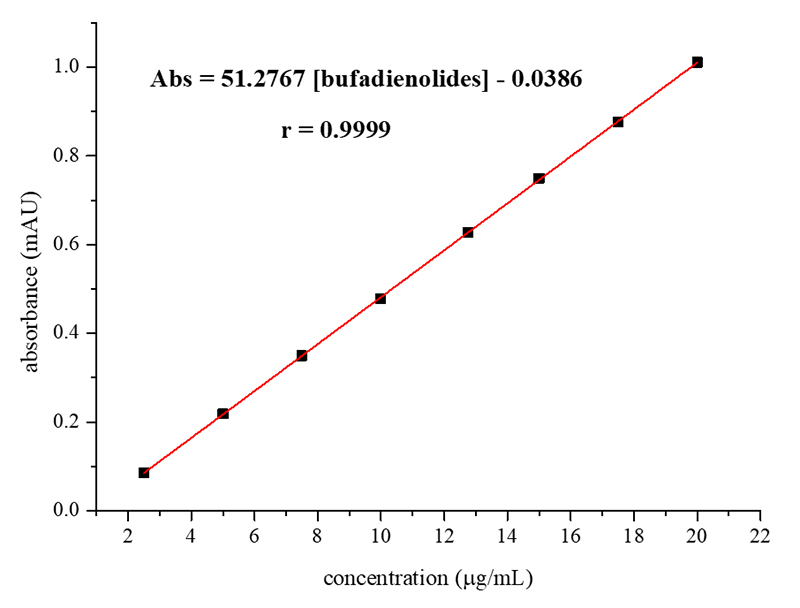
Analytical curve obtained after reaction of the marinobufagin standard solutions with excess of 5% aqueous NaOH solution (wavelength - 296 nm).

The selectivity of the developed method, evaluated by comparing the UV-Vis spectra of the extracts investigated before and after the reaction with the 5% sodium hydroxide solution, was quite satisfactory, clearly demonstrating a bathochromic shift (Figure 2). The constructed analytical curve, after the reaction of marinobufagin standard with the sodium hydroxide solution for quantifying the bufadienolides content in the PGS extracts, exhibited significant linearity (Figure 3) within the defined working range for this study (2.5-20.0 µg/mL), as evidenced by the correlation coefficient value obtained in the linear regression (r = 0.9999).

The linear regression analysis of the dataset revealed that the proposed method exhibited good sensitivity (S = 51.2767) and very low values for the limits of detection (LOD = 1.3 × 10-4 µg/mL) and quantification (LOQ = 3.9 × 10-4 µg/mL) of bufadienolides. The calculation of the relative standard deviation (%RSD) of eight replicates (independent experiments) was performed to assess the intraday precision (repeatability) of the method and taking into consideration that the calculated relative standard deviation (%RSD = 0.39) was lower than the threshold defined by the International Conference on Harmonization (%RSD ≤ 5.0), the developed method showed suitable intraday precision.

Similarly, the assessment of interday precision (reproducibility) of the method was conducted. This time, absorbance values of sixteen samples, prepared at the same concentration (25 µg/mL), were recorded on two consecutive days (eight readings each day). After determining the total bufadienolides content in the samples, expressed in the same unit of measurement as the intraday precision (mg of EqMB/g of extract), the calculation of the relative standard deviation (%RSD) was performed for the 16 toad PGS extracted solutions. The results are summarized in Table 2.

**Table 2.** Quantification of total bufadienolides in replicates of toad parotoid secretion extracts for evaluating the intraday and interday precision of the proposed UV-Vis spectrophotometric method.

Number of samples (n)	Total bufadienolides mean (mg EqMB/g of extract)	SD	RSD (%)
8	458.90	1.79	0.39
16	466.96	2.57	0.55

The recovery ranged from 84% to 99% in the three tested concentration levels, showing consistency with other studies reported in the literature [29, 30]. All analyses were conducted in triplicate. Table 3 presents the recovery rates of the marinobufagin standard obtained after the experimental assays were performed to verify the accuracy of the proposed UV-Vis method. Regarding all the robustness tests, the value of t_cal_ was lower than that of t_tab_, indicating that the means obtained before and after parameter alteration are statistically equivalent (p > 0.05). The results are summarized in Table 4.

**Table 3.** Recovery rate of the marinobufagin standard to evaluate the accuracy of the proposed method for quantifying total bufadienolides in toad SGP samples.

Standard addition (level)	Replica	Absorbance (mAU)	C_D_ (µg/mL)	Recovery (%)
Low (50 µg/mL) C_T_ = 2.5 µg/mL	S1	0.675	13.92	99.59
	S2	0.670	13.82	95.69
	S3	0.678	13.98	101.93
	**Mean**	**0.674**	**13.90**	**99.07**
Medium (100 µg/mL) C_T_ = 5.0 µg/mL	S1	0.789	16.14	94.26
	S2	0.799	16.33	98.16
	S3	0.794	16.24	96.21
	**Mean**	**0.794**	**16.24**	**96.21**
High (200 µg/mL) C_T_ = 10.0 µg/mL	S1	0.979	19.85	84.18
	S2	0.982	19.90	84.77
	S3	0.974	19.75	83.21
	**Mean**	**0.978**	**19.83**	**84.05**

C_D_: concentration calculated by the method for extracts spiked with standard solutions.; C_T_: theoretical concentration calculated for the standard in the extract solution (specific for each level); C_B_: concentration determined for the extracts not spiked with standard solutions (C_B_ = 11.43 µg/mL).

**Table 4.** Evaluation of the robustness of the proposed UV-Vis spectrophotometric method.

	Proposed conditions		Test 1		Test 2		Test 3		Test 4		Test 5	
Replica	Abs.	TB	Abs.	TB	Abs.	TB	Abs.	TB	Abs.	TB	Abs.	TB
S1	0.642	530.92	0.657	542.62	0.653	539.50	0.631	522.34	0.645	533.26	0.631	522.34
S2	0.639	528.58	0.661	545.74	0.659	544.18	0.640	529.36	0.642	530.92	0.637	527.02
S3	0.647	534.82	0.652	538.72	0.651	537.94	0.636	526.24	0.638	527.80	0.628	520.00
Mean	0.643	531.44	0.657	542.36	0.654	540.54	0.636	525.98	0.642	530.66	0.632	523.12
t_cal_			1.79		1.63		0.90		0.16		1.34	

Test 1 - altered parameter: base volume; Test 2 - altered parameter: base concentration; Test 3 - altered parameter: used base; Test 4 - altered parameter: reaction time; Test 5 - altered parameter: agitation time; TB - total bufadienolides (mg of EqMB/g of extract); Abs. - absorbance (mAU); t_tab_ = 2.78 (two-tailed test; degrees of freedom = 4; α = 0.05).

### Quantification of total bufadienolides in the extracts

**Figure 4.**
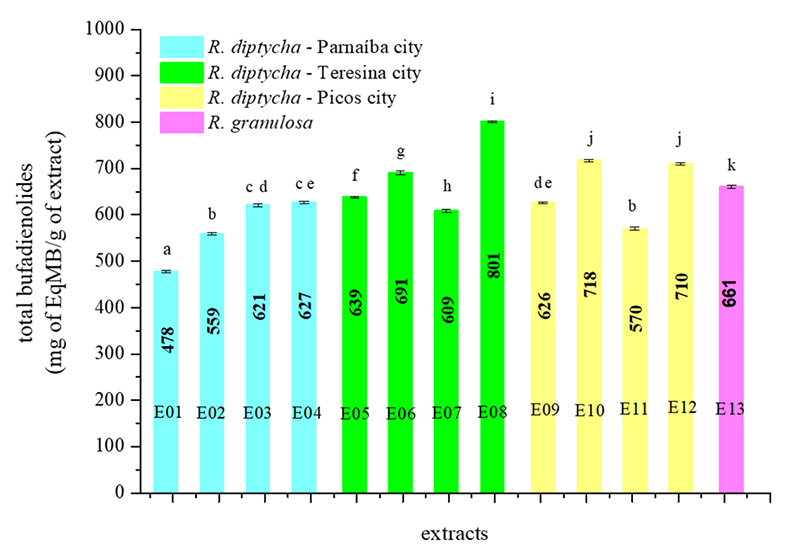
Quantification of total bufadienolides in paratoid secretions samples from toads (Rhinella genus) of the state of Piauí, Brazil.

Based on the recorded absorbance values and the constructed analytical curve, it was possible to quantify the total bufadienolides content in the 13 investigated PGS extracts, with the concentration expressed in milligrams of marinobufagin equivalents (MB) per gram of extract (mg of EqMB/g of extract) ± standard deviation. The total bufadienolides content in PGS extracts from 12 samples of R. diptycha ranged from 478 to 801 mg of EqMB/g of extract, while the R. granulosa sample presented 661 mg of EqMB/g of extract (Figure 4).

## Discussion

**Figure 5.**
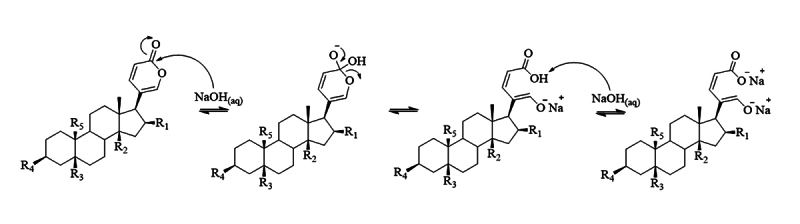
Nucleophilic acyl substitution reaction of the α-pyrone group of bufadienolides with excess 5% NaOH.

The validation of an analytical method aims to ensure that it is safe, reliable, and suitable for its intended purpose, thereby being a crucial aspect for ensuring analytical quality [31]. The proposed UV-Vis spectrophotometric method was based on the bathochromic shift induced by the acyl nucleophilic substitution reaction between the α-pyrone group of bufadienolides (296 nm) present in the toad extracts and the hydroxyl group from the 5% aqueous NaOH solution, after 60 minutes, generating a divalent anion as the final product, detected at 356 nm. The reaction observed in Figure 5 is specific to bufadienolides, and does not occur with alkaloids, arginine diacids, or biogenic amines, which are other constituents present in the matrix but lack the α-pyrone group (no bathochromic shift occurs).

The absorbance of eight replicates (solutions of PGS extract at a concentration of 25 µg/mL) was measured allowing for the determination of the total bufadienolides content in these samples, expressed in milligrams of marinobufagin (MB) equivalents per gram of extract (mg of EqMB/g of extract). Considering that the calculated value for the relative standard deviation (%RSD = 0.55) was lower than the value defined in the recommendations of the ICH (%RSD ≤ 5.0), the proposed method demonstrates satisfactory interday precision (reproducibility).

The promising results of the standard substance recovery assays ensured the accuracy of the developed spectrophotometric method and the robustness of the method was confirmed through the assessment of statistical results from the t-test for comparing the means of total bufadienolides content in the samples. The parameters evaluated were as follows: agitation and reaction times, volume, concentration, base substitution, agitation, and reaction time.

The developed method proved suitable for determining the total bufadienolides content in MeOH extracts of paratoid secretion from 12 samples of R. diptycha toads and one sample of R. granulosa. Observing the extracts of R. diptycha, the concentration of total bufadienolides in the extracts from PGS of male specimens was higher when compared to females. Considering the collection city from the secretions, the extract obtained from the PGS of toads of the city of Teresina exhibited the highest of total bufadienolides content among all the samples investigated.

Analogously, the extract of the paratoid secretions collected in the city of Parnaíba exhibited the lowest amount of these metabolites. Regarding the seasonal period in which the paratoid secretions were collected, a balance was observed in the total bufadienolides quantities determined in the extracts produced in both seasons.

According to statistical analysis based on the Tukey test (represented by the letters above the columns), the total bufadienolides contents determined in the samples of R. diptycha and R. granulosa are statistically different (p < 0.05). Considering only the R. diptycha extracts, the total bufadienolides content quantified in some samples from the cities Picos and Parnaíba was statistically equivalent (indicated by identical letters above the columns).

Thus, the developed method, based on the UV-Vis absorption spectrophotometry technique, is innovative as it enables the determination of total bufadienolides in toad paratoid secretions samples - an unprecedented approach. No existing methodology for this purpose is available in the current literature. It is also important to highlight that existing and widely used chromatographic techniques, particularly HPLC, do not allow for the quantification of total bufadienolides in samples but rather the specific (limited) determination of metabolites. In such cases, the use of high-cost standards is required, when commercially available [24, 32-34].

Considering that bufadienolides are the primary bioactive compounds in toad paratoid secretions and that research with these metabolites - aimed at identifying new bioactivities - has been expanding, the developed method can be strategically used as a preliminary analysis for selecting working matrices, optimizing time, and minimizing costs.

## Conclusion

This is the first proposed UV-Vis spectrophotometric method for determining the total bufadienolides content in samples of paratoid secretions from toads. The method demonstrated adequate linearity, detection limit, quantification limit, recovery rate, repeatability, and reproducibility, adhering to the recommendations of the International Conference on Harmonization (ICH). The observed bathochromic shift after the reaction of the toad extract solutions with NaOH confirmed the method's selectivity and its stability in the face of varying parameters, such as agitation and reaction times, volume, concentration, and substitution of the base used, ensuring its robustness. Therefore, the new developed method is innovative, simple, fast, accurate, robust, low cost, and can contribute to future research focused on the quantification of total bufadienolides in samples of anurans glandular secretions. In addition to serving as a strategic tool in the selection of work matrices, optimizing time, and minimizing costs.

### Abbreviations

AcOEt: ethyl acetate; CEUA: Ethics Committee on the Use of Animals; CHCJ: Herpetology Scientific Collection Jorge Jim; DAD: Diode Array Detector; EARD: ethyl acetate extract of the paratoid secretions of R. diptycha toads; ESI: electrospray ionization; HPLC: high-performance liquid chromatography; HRMS: high-resolution mass spectrometry; IBAMA: Brazilian Institute of Environment and Renewable Natural Resources; ICH: International Conference on Harmonization; LC-MS: liquid chromatography-mass spectrometry; LOD: limit of detection; LOQ: limit of quantification; MB: marinobufagin; MeCN: acetonitrile; mg of EqMB/g of extract: milligrams of marinobufagin equivalents per gram of extract; PGS: paratoid gland secretions; RSD: relative standard deviation; SD: standard deviation; SISBIO: Biodiversity Authorization and Information System; SISGEN: National System for the Management of Knowledge about Biodiversity; TB: total bufadienolides; TLC: thin layer chromatography; UV-Vis: ultraviolet-visible.

## Data Availability

All data generated or analyzed during this study are included in this article.

## Supplementary material

The following online material is available for this article:

Additional file 1. Thin layer chromatography of the isolated marinobufagin standard.

Additional file 2. Chromatogram and purity content of the isolated marinobufagin standard.

Additional file 3. UV-Vis spectrum of the isolated marinobufagin standard.

Additional file 4. Chromatographic data of the isolated marinobufagin standard. **(A)** Total ion chromatogram (TIC). **(B)** Extracted ion chromatogram (EIC) in the m/z 401.00 to 402.00 range. **(C)** High-resolution mass spectrum (HRMS - positive mode).

Additional file 5. Molecular structure with the exact mass of the marinobufagin substance (ChemDraw software) and calculated relative error value.
