# Supplementary material for: Development and validation of a spectrophotometric method for the quantification of total bufadienolides in samples of toad glandular secretions
Source: J Venom Anim Toxins Incl Trop Dis. 2025 May 16;31:e20240064. doi: 10.1590/1678-9199-JVATITD-2024-0064 (PMC12092071; doi:10.1590/1678-9199-JVATITD-2024-0064)
Supplement: Additional file 2. [file 1678-9199-jvatitd-31-e20240064-s2.pdf]

J Venom Anim Toxins incl Trop Dis
https://doi.org/10.1590/1678-9199-JVATITD-2024-0064

Supplementary Material to "Development and validation of a spectrophotometric method for the quantification of total bufadienolides in samples of toad glandular secretions"

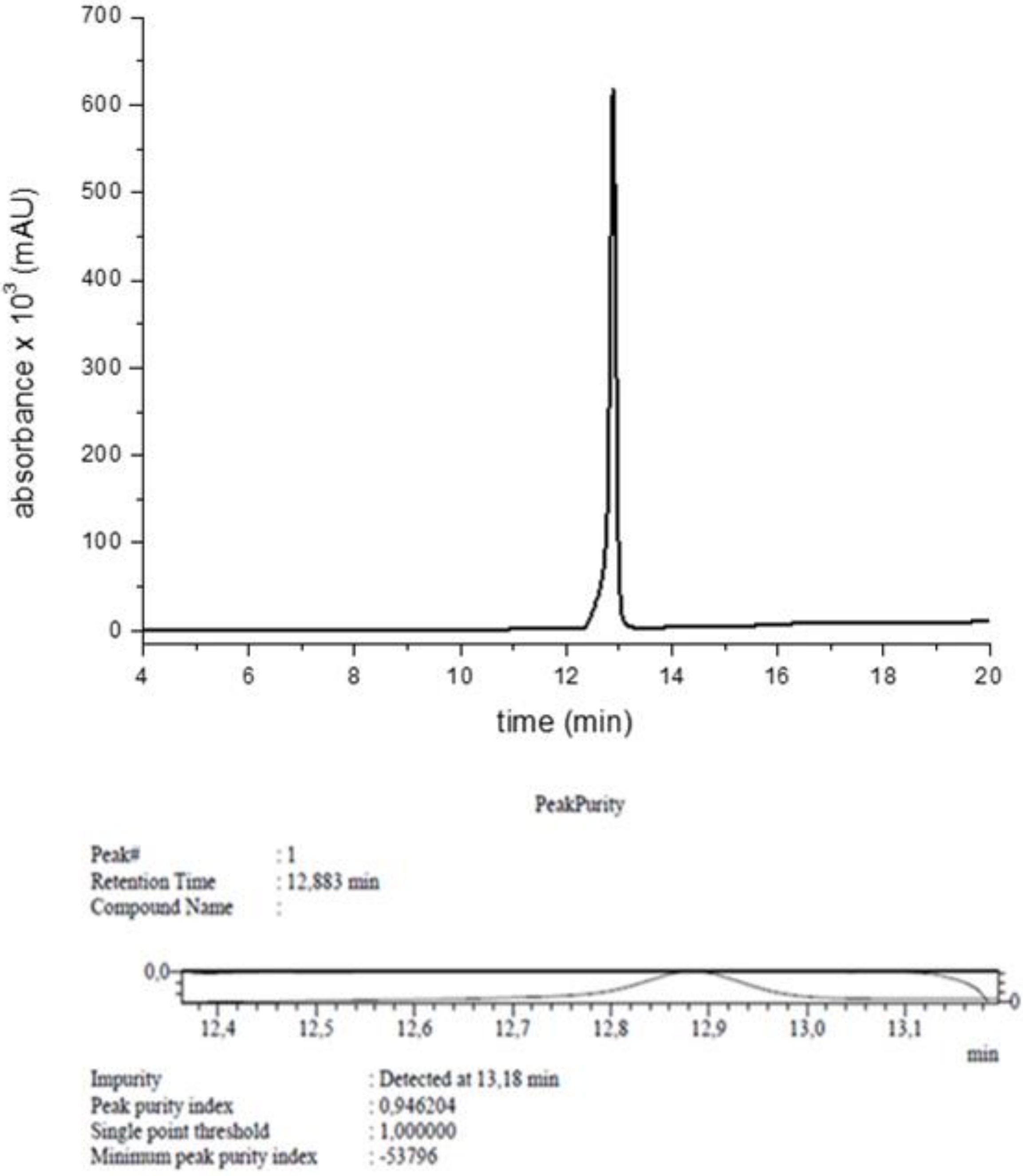

Additional file 2. Chromatogram and purity content of the isolated marinobufagin standard.
