# Supplementary material for: Development and validation of a spectrophotometric method for the quantification of total bufadienolides in samples of toad glandular secretions
Source: J Venom Anim Toxins Incl Trop Dis. 2025 May 16;31:e20240064. doi: 10.1590/1678-9199-JVATITD-2024-0064 (PMC12092071; doi:10.1590/1678-9199-JVATITD-2024-0064)
Supplement: Additional file 5. [file 1678-9199-jvatitd-31-e20240064-s5.pdf]

*J Venom Anim Toxins incl Trop Dis*
https://doi.org/10.1590/1678-9199-JVATITD-2024-0064

# Supplementary Material to "Development and validation of a spectrophotometric method for the quantification of total bufadienolides in samples of toad glandular secretions"

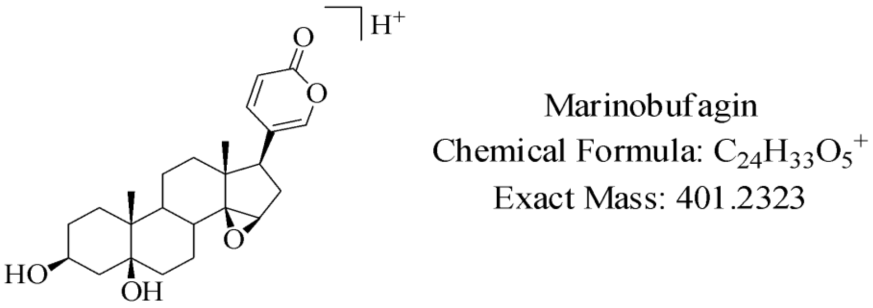


| Compound | Molecular formula | $[M+H]^+$ Theoretical | $[M+H]^+$ Measured | Error (ppm) | References |
|---|---|---|---|---|---|
| Marinobufagin | $C_{24}H_{32}O_5$ | 401.2323 | 401.2325 | 0.50 | [24]<br>[25]<br>[26] |

**Additional file 5.** Molecular structure with the exact mass of the marinobufagin substance (ChemDraw software) and calculated relative error value.
